# Tablet-based self assessment memory scale-revised (SAMS-R) evaluates memory functions for older adults

**DOI:** 10.3389/fnagi.2024.1512947

**Published:** 2025-01-22

**Authors:** Hisatomo Kowa, Ryoko Kumagai, Yutaro Oki, Miki Imamura, Yuka Suzuki

**Affiliations:** ^1^Kobe University Graduate School of Health Sciences, Kobe, Japan; ^2^OMRON Healthcare Co., Ltd., Kyoto, Japan

**Keywords:** digital cognitive assessments, memory function, WMS-R logical memory, mild cognitive impairment, Alzheimer’s disease

## Abstract

**Background:**

The demand for more accurate and early diagnosis of mild cognitive impairment (MCI) patients due to Alzheimer’s disease (AD) has increased after disease-modifying drugs were launched. Among these needs, there is a requirement for tools that can easily assess the ability to recall memories, which changes early in the disease.

**Objectives:**

We established Self Assessment Memory Scale (SAMS) method before, which includes 8-picture recall test and 16-word recognition test. We adopted this method to software that can be operated on a tablet computer so that participants can perform the method independently. The purpose of this study was to validate this method.

**Design:**

Cross sectional research.

**Setting:**

Some of the participants were recruited from hospitals for patients diagnosed with AD or MCI. The others were recruited from three regional cohorts of healthy older adults.

**Participants:**

The total number of participants was 304 (20 of whom had AD or MCI), and the mean age was 71.2 years. 64% of the participants were women.

**Measurements:**

We used the logical memory subtest of the WMS-R as the standard for memory evaluation and assessed the relationship between this score and the SAMS score calculated by the software.

**Results:**

The 2nd SAMS score were higher than the 1st SAMS score in some participants, on the other hand, the intraclass correlation coefficient was good. Since the number of false recognition in the 16-word recognition test was higher in participants with lower LM II scores, we developed a new score to reflect the ratio of false recognition, SAMS-R, and we observed it has good correlation with LM II. The mean SAMS-R score decreased gradually after the age of 65 years, indicating that age-related changes in memory recall can be detected. The ROC curve analysis was conducted to evaluate the detectability to determine whether if the WMS-R LM II score is above or below 10, showing that the AUC was greater than 0.9.

**Conclusion:**

SAMS-R, which can be performed on a tablet literally by himself/herself independently, shows a high correlation with the WMS-R Logical Memory II score, and has the advantage of being performed in a short time without the need for a clinical psychologist or other personnel.

## 1 Introduction

The number of people living with dementia worldwide is estimated at 55.2 million in 2019 and is expected to increase to 139 million by 2050 (World Health Organization, 2021). Japan has the highest aging rate in the world, and it is estimated that one in four older adults will have dementia or mild cognitive impairment (MCI) by 2025 (Ninomiya, 2024), making it a serious social problem. One solution to this problem is to try to prevent dementia. We recently conducted a randomized controlled trial of an 18-month multifactorial intervention in older adults at risk of developing dementia and found that the intervention group significantly maintained or improved cognitive function (Oki et al., 2024). The other solution is through drug therapy. For Alzheimer’s disease (AD), the most frequent cause of dementia, disease-modifying drugs called Lecanemab (Cummings et al., 2023; van Dyck et al., 2023) and Donanemab (Sims et al., 2023) were approved by the FDA and PMDA (Pharmaceuticals and Medical Devices Agency) in 2023 and 2024 for the removal of senile plaques, one of the characteristic pathological structures of AD. As these therapies are not effective once symptoms have progressed, early diagnosis is even more important to maximize the effectiveness of treatment (Silva et al., 2019).

One of the earliest symptoms of AD is impaired memory recall. The WMS-R Logical Memory Test has been used as a method to accurately assess memory function (Yamaguchi et al., 2021), but it takes more than 30 min and is not suitable as a screening test. We developed the Self Assessment Memory Scale (SAMS), which correlates well with the WMS-R Logical Memory Test II score (Kowa et al., 2022). It is a simple method that can be completed in about 10 min and is a tool for assessing early memory impairment.

As this method is expected to be used in the future at home and community check-ups rather than in hospitals, a method that can be carried out by himself/herself alone on a tablet computer was considered necessary. In addition, in the previous version (SAMS), if a participant answers “yes” even when the word is not shown (i.e., false recognition), it would not be counted as “incorrect answer,” and thus false recognition could not be assessed when the participant answers “yes” to all questions. To solve this issue, we developed a new method with which false recognition can be assessed (called SAMS-R). In this article, we examined whether the SAMS-R on a tablet computer shows a good correlation with the WMS-R, as in the previous paper (Kowa et al., 2022).

## 2 Methods

### 2.1 Participants

The participants of this study were recruited from five cohorts (Table 1), two of those were patients with AD and of MCI diagnosed clinically based on the NIA/AA Guidelines for Diagnosis of AD (Wechsler, 1987) and MCI (Wechsler, 2009) at the Department of Neurology, Kobe University Hospital (Cohort A) and at the Kusunoki Clinics (Cohort B). Cognitively normal participants were recruited among healthy volunteers living in Kobe City (Cohort C), recruited through websites by Omron Health Care (Cohort D) or come of the participants of J-MINT PRIME Tamba study (Cohort E) (Table 1). Totally, 304 people have been included in this study.

**Table 1:** Participant demographics.

Cohort name	A	B	C	D	E
Number of participants	7	13	118	24	142
Number of female participants (%)	2 (28.6)	6 (46.2)	80 (67.8)	1 (4.17)	106 (74.6)
Age mean ± SD	77.7 ± 2.9	81.5 ± 4.9	62.5 ± 7.8	58.3 ± 9.7	75.3 ± 4.6

### 2.2 SAMS software

This test on software consists of 2 tasks, one is for 16-word recognition task and the other is for 8-picture recall task. The detail of both tasks is shown in previous paper (Kowa et al., 2022). Each task is composed of two parts: one was to present the words to be memorized, i.e., the memorization part, and the other was to check whether the words were actually memorized, i.e., the recall part. We set a time limit for recall part of 16-word recognition task (each response is 10 s) and of 8-picture recall task (10 min).

In between the two parts, the participants played a game with an interval of 75 s.

Some participants were administered the test twice; for the second SAMS test, participants in Cohort E were administered 1 week later using the same words.

For participants in cohorts A and B, the test was administered on the same day as the first test, using a different set of words.

### 2.3 SAMS-R score

For overcoming the issue of false recognition, we have modified the original SAMS formula; added the ratio of correct responses to the 8-picture recall, that of 16-word recognition task, and subtracted false recognition, which counts minimum −1.0 and a maximum of 2.0 for the percentage of correct answers, which we call SAMS-R (Self-Assessment Memory Scale-Revised).

### 2.4 Statistical analysis

The association between 8-picture recall and LM II, 16-word recognition and LM II, and the SAMS-R score and LM II were analyzed by Spearman’s correlation coefficient.

For each cohort participant’s scores across age groups, continuous variables were compared by Kruskal-Wallis test, and categorical variables were compared between groups by Fisher’s exact test. The Tukey method was used to correct for multiple comparison.

All statistical analyses were performed using JMP 18.0.1, and the significance level was set at less than *p* value 0.05 with a two-tailed test.

### 2.5 Ethics review committee

This study was approved by the Ethics Committee of the Graduate School of Health Sciences, Kobe University, the Ethics Committee of the Graduate School of Medicine, Kobe University, the Ethics Committee of Yoyogi mental clinic and the Ethics Committee of Omron Healthcare.

## 3 Results

### 3.1 8-picture recall test

**Figure 1:**
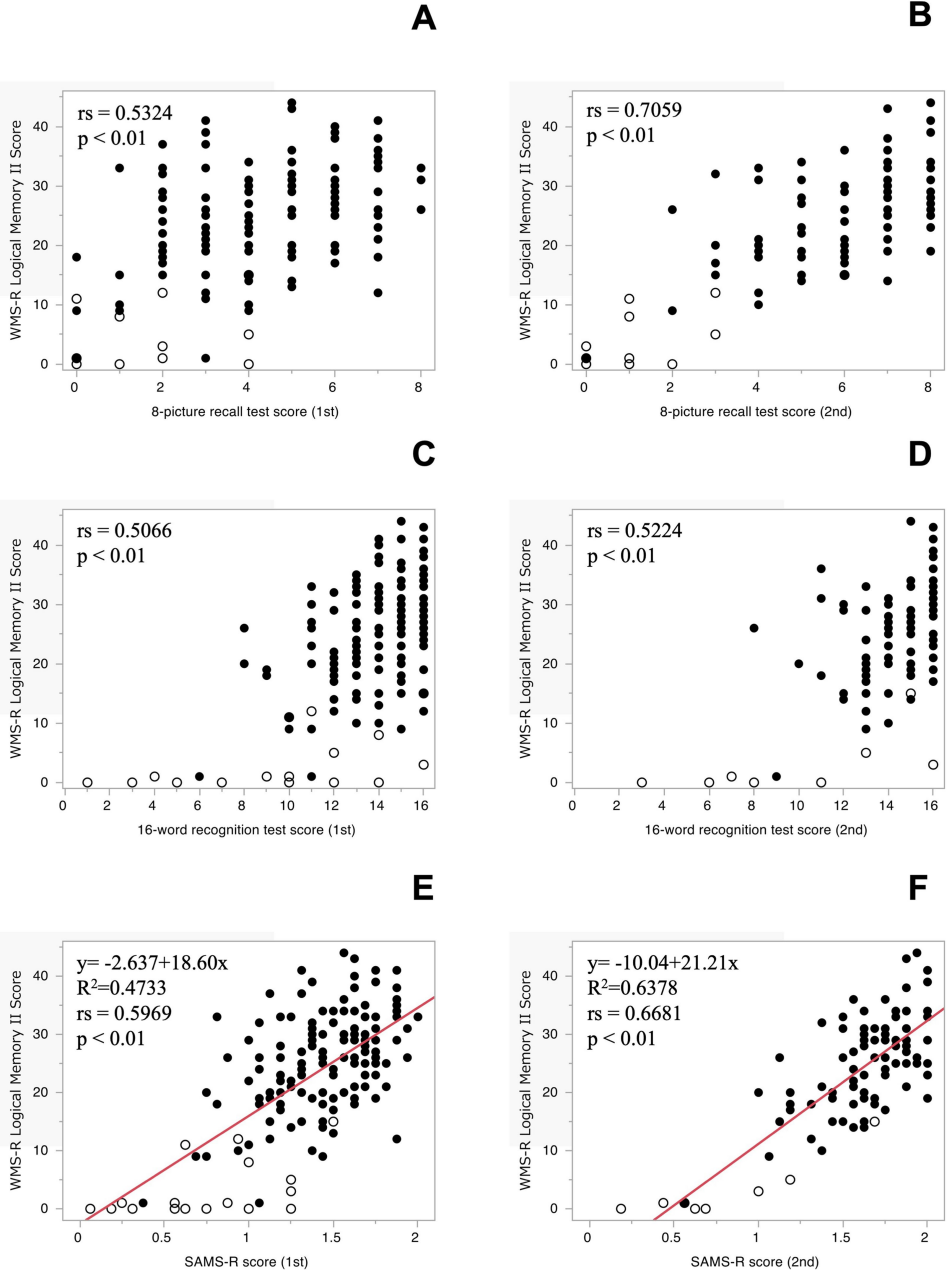
The correlation between the WMS-R Logical Memory II and 8-picture recall test [1st **(A)**, 2nd **(B)**], 16-word recognition test [1st **(C)**, 2nd **(D)**], respectively. The regression analysis between the WMS-R Logical Memory II and SAMS-R Score [1st **(E)**, 2nd **(F)**] and both lines show the equation of a regression. ○: Cognitively normal participants, •: Cognitively abnormal participants.

Correlations between the results of the first and second tests of 8-picture recall, one of the two SAMS tests, and the LM II were determined, respectively. The results showed that the first test was significantly associated with LM II with rs = 0.5324 (*p* < 0.01) (Figure 1A) and the second test with rs = 0.7059 (*p* < 0.01) (Figure 1B).

### 3.2 16-word recognition test

The correlations between the results of the first and second original 16-word recognition test, another test of the SAMS, and the LM II were then determined. The results showed that the first test was significantly related to LM II with rs = 0.3990 (*p* < 0.01) and the second test with rs = 0.4175 (*p* < 0.01), but the correlation coefficient was lower than expected. The correlation coefficient was lower than expected, since the number of false-reconfirmation questions, i.e., wrong answers that were not correct, was not reflected in this figure. False recognition tended to be higher in participants with lower LM II scores in both the first and second trials (data not shown). When the number of false recognitions was subtracted from the number of correct responses, the correlation with LM II was improved 1st test: rs = 0.5066 (*p* < 0.01), 2nd test: rs = 0.5224 (*p* < 0.01) (Figures 1C,D).

### 3.3 SAMS-R score

The correlation between LM II and the sum of the percentage of correct responses in the 8-picture recall test, using the new score that took into account false recognition in the 16-word recognition test, were calculated: first time: rs = 0.5969 (*p* < 0.01, *n* = 162), second time: rs = 0.6681 (*p* < 0.01, *n* = 101). After checking the residuals for normality, we conducted regression analyses. The regression analysis showed coefficient of determination of first time: *R*^2^ = 0.4733 (*p* < 0.01), second time: *R*^2^ = 0.6378 (*p* < 0.01) (Figures 1E,F). Before When we calculated SAMS using the previous formula, the correlations between the first and second SAMS score and LM II were rs = 0.5765 (*p* < 0.01, *n* = 162) and rs = 0.7243 (*p* < 0.01, *n* = 108).

**Figure 2:**
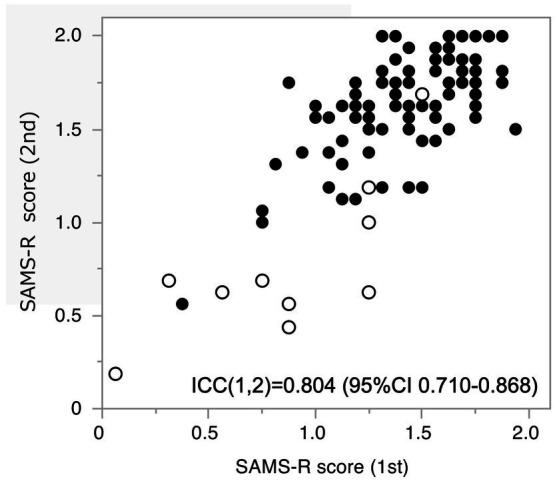
Intraclass correlation coefficient between the 1st and the 2nd score of SAMS-R. ○: Cognitively normal participants, •: Cognitively abnormal participants.

Therefore, we named this score as SAMS-R and used it in this study. And then we also assessed the SAMS-R using the exact same word set. The correlation of LM II and 1st SAMS-R was rs = 0.4905 (*p* < 0.01, *n* = 147) and 2nd SAMS-R was rs = 0.6272 (*p* < 0.01, *n* = 97). We observed the 2nd SAMS-R score were higher than 1st SAMS-R score in some participants, but the Intraclass correlation coefficient was a satisfactory result, ICC(1,2) = 0.804 (95%CI 0.710–0.868), (Figure 2).

### 3.4 SAMS-R standard values by age group

**Figure 3:**
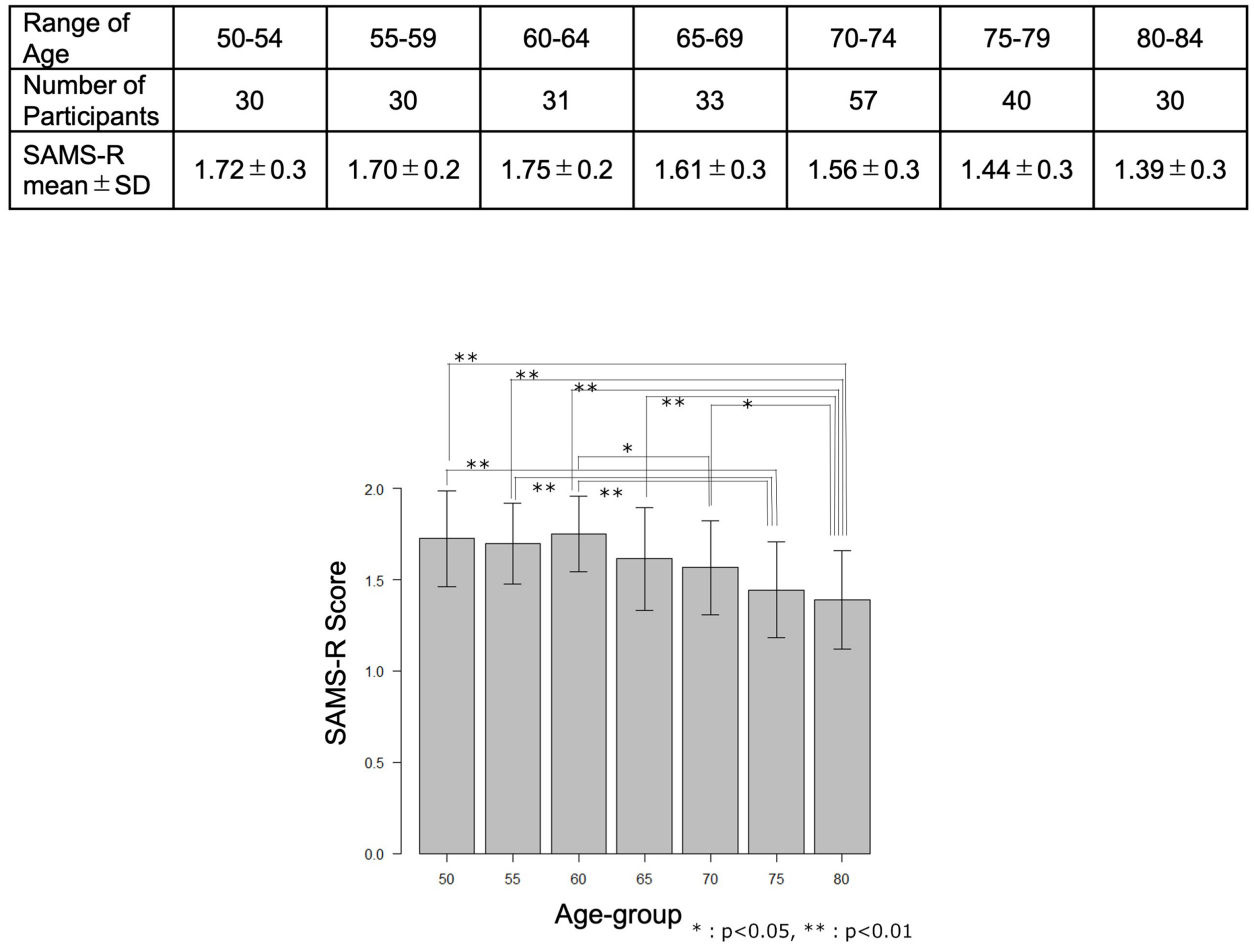
The mean values and standard deviations for each 5-year age group.

The mean values and standard deviations for each 5-year age group are shown in Figure 3, which indicate that the mean value tends to decrease gradually after the age of 60, suggesting that this method may be able to detect the age-related decline in memory recall.

### 3.5 ROC analysis

**Figure 4:**
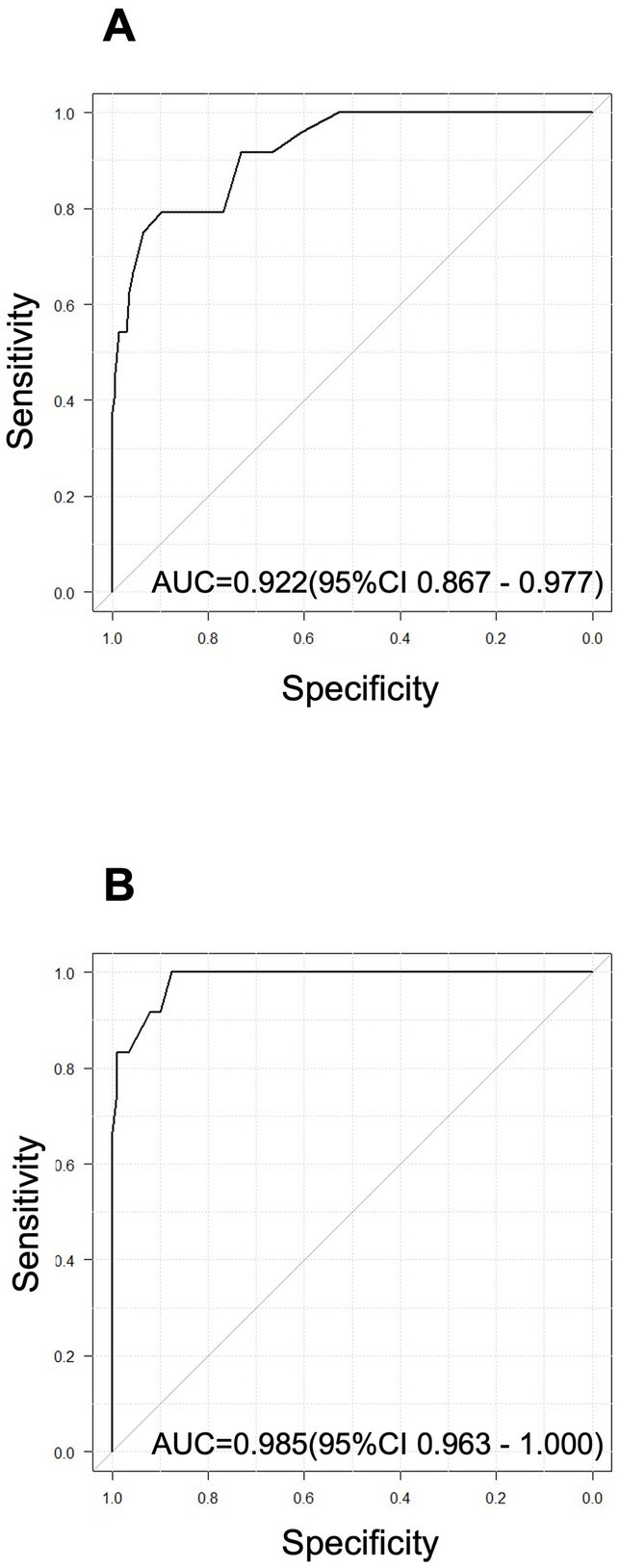
ROC analysis for SAMS-R [1st **(A)** and 2nd **(B)** test, respectively] to detect the WMS-R LM II score above or below 10.

Here, we conducted the receiver-operating-characteristic (ROC) curve analysis for SAMS-R (1st and 2nd test, respectively) to detect the WMS-R LM II score above or below 10. The AUC (area under the curve) in the 1st test and 2nd test were 0.922 (95%CI 0.867–0.977, *p* < 0.01) and 0.985 (95%CI 0.963–1.000, *p* < 0.01), respectively (Figures 4A,B).

## 4 Discussion

We developed software that allows a single subject to perform the SAMS method we reported on a tablet terminal. The evaluation of its performance using this software confirmed a strong correlation with the logical memory II score of the WMS-R, as mentioned in the previous presentation. To put it simply, the software method that can quickly assess memory recall ability without the use of psychologists or other assessors has been completed.

The SAMS score, which consisted of a recall of 8 pictures and a recognition of 16 words, improved the second time in the some participants (but the correlation was satisfactory). The reason for this improvement is likely due to the fact that older individuals are unfamiliar with the operation of digital tools when they first use them (Kuerbis et al., 2017). This is why it is recommended to use the second time score as the ability value. It is necessary to observe how the score changes after the third and subsequent iterations when implementing the evaluation with this software in society in the future.

As we described above, it was confirmed that false recognition was more frequent in participants with low scores on LM II, so the new SAMS-R reflecting the ratio of false recognition was defined and its correlation with LM II was examined. Because this method indicated a better correlation, it was decided to adopt the SAMS-R.

The mean value of the SAMS-R in a population of normal cognitive participants decreased gradually after the age of 65 years, and the normal value of WMS-R and WMS-IV logical memory II also changed with age (Wechsler, 1987, 2009), indicating that, in addition to the high correlation to LM II, the SAMS-R can also detect changes in the gradual decline of memory recall with age. The next step is to follow up a single subject to see if a similar process can be detected.

In the present study, we investigated whether the SAMS-R could identify abnormal values in the WMS-R logical memory test by using the ROC curve, and found that the AUC was 0.985, which was an extremely high value. This result ensures the effectiveness of this method. The test requires shorter time than the logical memory test and can be carried out by one person alone with tablet software.

In a variety of situations, it can be used as a simple test to identify the initial clinical manifestation of Alzheimer’s disease, which is an impaired recall. For example, even non-dementia specialists can assess the severity of a patient’s complaints of forgetfulness, and combining this test with a plasma biomarker of amyloid in the brain, which is currently under development (Scheltens et al., 2016), may enable rapid and accurate diagnosis of patients who are candidates for Lecanemab (van Dyck et al., 2023) or Donanemab (Sims et al., 2023). Future research should examine the relationship between actual amyloid positivity and the SAMS-R score.

Currently in Japan, similar tablet-based cognitive function assessment tools such as Cogstate Brief Battery (Maruff et al., 2013), Cogevo (Ichii et al., 2020) and MIREVO (Oyama et al., 2019) exist. The main purpose of these tools is to evaluate general cognitive function, and therefore, they have few assessment tests to evaluate memory and cannot detect changes in memory at a very early stage. On the other hand, the software we have developed is specialized for memory and can detect cognitive decline at an earlier stage. In the future, it is expected that combining SAMS-R with other cognitive tests will enable a more detailed assessment of cognitive functions.

The study’s limitation was the small number of participants in the low cognitive function group. The high proportion of female participants is also a limitation of this paper. We could not analyze the normal score of the SAMS-R for each age group, because the number of participants was small in each group. Moreover, we need several studies in the future to obtain longitudinal data. To achieve this, we have to prepare multiple word patterns with similar difficulty levels.

We have developed and established the SAMS-R, which can be performed on a tablet literally by himself/herself, showing a high correlation with the WMS-R Logical Memory II, and has the advantage of being performed in a short time without clinical psychologist or other personnel. Our goal is to implement the SAMS-R and make it a tool that can contribute to screening and early diagnosis of Alzheimer in the community.

## Data Availability

The raw data supporting the conclusions of this article will be made available by the authors without undue reservation.
